# EphA4 Negatively Regulates Myelination by Inhibiting Schwann Cell Differentiation in the Peripheral Nervous System

**DOI:** 10.3389/fnins.2019.01191

**Published:** 2019-11-13

**Authors:** Ruyue Chen, Xiaoming Yang, Bin Zhang, Shengran Wang, Shuangxi Bao, Yun Gu, Shiying Li

**Affiliations:** ^1^Jiangsu Key Laboratory of Neuroregeneration, Co-innovation Center of Neuroregeneration, Nantong University, Nantong, China; ^2^Jiangsu Clinical Medicine Center of Tissue Engineering and Nerve Injury Repair, Affiliated Hospital of Nantong University, Nantong, China

**Keywords:** EphA4, Schwann cells, myelination, negative regulation, nerve injury

## Abstract

Myelin plays a crucial role in axon function recovery following nerve damage, and the interaction between Schwann cells (SCs) and regenerating axons profoundly affects myelin formation. Eph receptor A4 (EphA4), a member of the Eph tyrosine kinase receptor family, regulates cell-cell interactions via its ligand ephrins. However, our current knowledge on how EphA4 regulates the formation of myelin sheaths remains limited. In order to explore the roles of EphA4 in myelination in the peripheral nervous system, we used a combination of (1) a co-culture model of dorsal root ganglion (DRG) explants and SCs, (2) a SC differentiation model induced by db-cAMP, and (3) a regeneration model of crushed sciatic nerves in rats. Our results demonstrated that EphA4 inhibited myelination by inhibiting SC differentiation and facilitating SC proliferation *in vitro*. The *in vivo* experiments revealed that EphA4 expression in SCs is upregulated following nerve crush injury and then downregulated during remyelination. Moreover, silencing of EphA4 by siRNA or overexpression of EphA4 by genetic manipulation can accelerate or slow down nerve remyelination in crushed sciatic nerves. Taken together, our results suggest that EphA4 may negatively regulate myelination by abrogating SC differentiation.

## Introduction

Myelin is a lamellar structure formed by glial cells entangled with neuronal axons, mainly composed of oligodendrocytes in the central nervous system (CNS) and Schwann cells (SCs) in the peripheral nervous system (PNS). Myelinated axons require support from local glial cells to maintain both the long-term survival of axons and regenerative neurological integrity (Griffiths, 1998; Nave, 2010; Nave and Werner, 2014; Nicolas, 2017). Clinically, myelin abnormalities affect nerve impulses, leading to the degradation of axonal lesions, thereby causing neurological diseases, including nervous system inflammatory demyelinating disease, senile dementia, schizophrenia, and progressive neurogenic tibial muscular atrophy (Seal et al., 2008; Pereira et al., 2012; Cantoni et al., 2015; Marcin et al., 2015; Mcgavern et al., 2015). Therefore, investigating the process of myelination is of great relevance to the treatment of diseases of the nervous system, especially demyelinating diseases.

Myelination in the PNS involves a complex interaction between SCs and axons. Axon-derived signals initiate myelination by regulating glial cell survival and proliferation and inducing glial cell differentiation. Similarly, glial cells also provide signals to neurons during myelination, cytoskeletal reorganization of axons, organization of organelle content, and modulation of the rate of axonal transport (Barateiro and Fernandes, 2014; Taveggia, 2016). Eph/Ephrin is an important mediator of bidirectional signaling between axons and glial cells in the nervous system, involved in multiple processes such as astrocyte activation and scar formation, axon guidance, axonal regeneration, synaptic plasticity, and myelination (Linneberg et al., 2015). EphA4, a member of the Eph tyrosine kinase receptor family, binds two subfamily members, EphrinA and EphrinB, to regulate various biological cellular functions (Liu et al., 2006; Pasquale, 2008). Studies have recently shown that EphA4 and EphrinB3 knockout mice show differences in myelination (Iwasato et al., 2007). Other research reported that genetic as well as pharmacological inhibition of EphA4 signaling increases survival in a disease modifier of amyotrophic lateral sclerosis (ALS) (Van Hoecke et al., 2012). In addition, activating Ephrins in zebrafish neuronal axons can induce EphA4 expression in oligodendrocytes, which affects the contact between axons and glial cells, thereby inhibiting myelin formation (Harboe et al., 2018). These studies suggest a role for EphA4 in regulating myelination in both the CNS and PNS. However, the mechanisms by which EphA4 regulates formation and maintenance of myelin sheaths remain poorly understood.

Here, we aim to identify the role of EphA4 in Schwann cell myelination and, in particular, to understand how it affects the myelinating capacity of SCs. We found that EphA4 expression, as assessed by Western blotting and immunostaining, is associated primarily with SCs in the nerve, neuron body in the DRG and, to a lesser extent, axons, both before and after peripheral nerve injury (PNI). Moreover, EphA4 abrogates SC differentiation and further SC myelination *in vitro* and inhibits remyelination of the regenerated axons in crushed sciatic nerves.

## Materials and Methods

### Animals, Surgical Procedure, and Virus Infection

The experimental procedures involving laboratory animals were performed according to the institutional guidelines of animal care of Nantong University, and approved by the Administration Committee of Experimental Animals, Jiangsu Province, China.

The surgical procedure was conducted as previously described (Gu et al., 2018). Fifty adult male Sprague Dawley (SD) rats weighing 200–250 g were provided by the Experimental Animal Center of Nantong University. They were anesthetized by intraperitoneal injection of 3% sodium pentobarbital solution (30 mg/kg body weight) prior to surgery. The sciatic nerve was exposed by making a skin incision and splitting apart the underlying muscles in the left lateral thigh, crushed at the level of the sciatic notch for 30 s using a fine hemostat. In sham rats, the sciatic nerves were exposed but not injured, and then incision were sutured. Following the sciatic nerve crush surgery, the sciatic nerve injections were performed immediately as recently described (Su et al., 2019). The replication-deficient *EphA4* gene carrying LV vector and control LV vector (LVCON195) were generated by Genechem (Shanghai, China) and the 5′-Chol-modified siRNA were provided by Ribobio (Guangzhou, China), as shown in Supplementary Table 1.

### Cell Culture

SCs were obtained as previously described (Gu et al., 2014). Briefly, enzymatically dissociated sciatic nerves excised from SD rats (1–3 d-old) were cultured at 37°C in 5.0% CO_2_ in Dulbecco's modified Eagle's medium (DMEM) supplemented with 10% fetal bovine serum (FBS) and penicillin/streptomycin on poly-L-lysine (PLL, Sigma, CA, USA) coated dishes. On the following day, 2 mM cytosine arabinoside (Sigma, CA, USA) was added to allow for cell incubation for an additional 24 h to remove fibroblasts, after which the medium was replaced with a complete medium containing 2 ng/mL NRG (R&D, MN, USA) and 2 mM Forskolin (Sigma, CA, USA) to stimulate cell proliferation. When cells covered 90% of the dish surface, they were incubated with anti-Thy1 antibody (1:1000, AbD Serotec, Raleigh, NC) on ice for 2 h, and with complement (Jackson Immuno, West Grove, PA) for another 1 h. All media and supplements were obtained from Gibco-Invitrogen (Carlsbad, CA, USA).

DRG neurons were generated as previously described (Päiväläinen et al., 2008; Gu et al., 2018) and embryonic DRG neurons were isolated from E14 rats and seeded onto 24-well plates which had been pre-coated with PLL. The neurons were cultured for 2 days in neuralbasal medium (Gibco) containing 2% B27 (Gibco), 1% penicillin-streptomycin (Sigma), 2 mM glutamine (Sigma), 10 μM uridine (Sigma), 10 μM 5-fluoro-2'-deoxyuridine (Sigma), and 50 ng/mL nerve growth factor (NGF, R&D, MN, USA). They were next cultured with neuralbasal medium in the presence of 2% B27, 2 mM glutamine, and 50 ng/mL NGF. To remove contaminating cells, the cultures were pulsed for 3 alternate rounds, each lasting 2 days, until DRG tissue consisted of sun-like divergence axons with nearly no other cells.

### Myelination Model *in vitro*

Myelinating co-cultures of SCs and DRG neurons were generated as previously described (Päiväläinen et al., 2008; Su et al., 2016). Purified SCs were added at a density of 1 × 10^5^ cells/ml to each DRG culture in 24-well plastic culture plates and cultured with DMEM containing 10% FBS and 50 ng/ml NGF. They were allowed to attach, after which the medium was changed to a DRG growth medium (Neuralbasal, 2% B27, 2 mM glutamine, and 50 ng/mL NGF) for 2 days. The DRG/SC co-cultures were further maintained for 4–6 days in the DMEM supplemented with ITS (1:100, Sigma), 0.2% BSA, and 50 ng/ml NGF. Fresh co-culture media supplemented with factors was changed every 2 days for a total of 3 times. To induce myelination, the media was replaced with myelination medium (DMEM, 15% FBS, 50 mg/ml ascorbic acid, 50 ng/ml NGF) and changed every 2 days until myelination was achieved in the following 12–28 days.

### Cell Proliferation Assay

The Cell-Light^TM^ EdU DNA Cell Proliferation Kit (Ribobio) was used to measure the proliferation rate of SCs as previously described (Gu et al., 2018). Approximately 40,000 SCs were plated onto the fasciculated axons or PLL-coated cover slips in DMEM containing 10% FBS for 1 day, after which the medium was changed to a non-proliferating medium (DMEM, 1% FBS) for another day. The proliferation rate of SCs was then determined according to the manufacturer's instructions. Briefly, 50 μM EdU was added to the cell medium and cells were incubated for 24 h, after which they were fixed with 4% formaldehyde in phosphate buffered saline (PBS) for 30 min. EdU labeling was next conducted according to the manufacturer's instructions. Hoechst 33342 (1 μg/ml, Sigma) was also applied for cell nucleus staining. Images were captured with a DMR fluorescence microscope (Leica Microsystems, Bensheim, Germany), and the percentage of cell proliferation was analyzed with 9 randomly selected fields from 3 different wells (3 fields/well), and repeated experiment 10 times.

### Cell Migration Assay

SC migration was assessed using a transwell-based assay as previously described (Parrinello et al., 2010). In brief, SCs were seeded onto the upper chamber of a transwell insert with 8 μm pores (Costar, Cambridge, MA) at a cell density of 1 × 10^6^ cells/mL in DMEM. To evaluate SC migration, the cells were allowed to migrate at 37°C in 5% CO_2_ for 24 h, after which the transwell insert was removed and the upper surface of its insert membrane cleaned with a cotton swab. The cells on the bottom surface of the insert membrane were next stained with 0.1% crystal violet for 30 min at room temperature and subsequently imaged and counted under a DMR inverted microscope (Leica).

### Cell Differentiation Assay

Schwann cell differentiation was induced as previously described, with minor modifications (Arthur-Farraj et al., 2011; Gu et al., 2018). Approximately 40,000 SCs were plated onto 24-well plastic culture plates with PDL-coated cover slips in DMEM containing 10% FBS for 24 h. They were then induced with DMEM/F12 plus 1% FBS, 1 mM db-cAMP (Sigma) and 20 ng/ml HRG for 3 days to acquire a differentiated phenotype. Western blotting was carried out to analyze the expression of protein markers for myelinating/non-myelinating SCs, and cell morphology was analyzed by immunocytochemistry.

### EphA4 siRNA Transfection

Purified SCs were seeded at a density of 3 × 10^6^ cells/ml in 24-well plates and cultured for 24 h, after which they were transfected with siRNAs for EphA4 and non-targeting negative control (Ribobio, Guangzhou, China) using lipofectamine RNAiMAX transfection reagent (Invitrogen, Carlsbad, CA) according to the manufacturer's instructions.

### RT-PCR

Total RNA was extracted using a Trizol reagent following the manufacturer's instructions (Invitrogen) and cDNA was synthesized using a cDNA Reverse Transcription Kit (Takara Biosystems, Kusatsu, Japan). Real-time quantitative PCR (RT-qPCR) was performed using SYBR Green Premix Ex Taq (TaKaRa) on a 7300 Real-Time PCR System (Applied Biosystems, Foster City, CA). The primers used in this experiment are listed in Supplementary Table 2. All reactions were conducted in triplicate, and the relative mRNA expression levels were calculated using the comparative 2^−ΔΔCt^ method and normalized against GAPDH.

### Western Blotting

Protein samples were prepared as previously described (Su et al., 2019), after which they were separated by 10% sodium dodecyl sulfate-polyacrylamide gel electrophoresis (SDS-PAGE). Following the transfer, the blots were probed with primary antibodies at 4°C overnight, further incubated with HRP-conjugated secondary antibody, and detected using a SuperSignal West Pico Chemiluminescent Substrate kit (Pierce). Chemiluminescence was revealed by Bio-Rad ChemiDoc XRS for 1–5 min and processed for data analysis using PDQuest 7.2.0 software (Bio-Rad).

The above-mentioned primary antibodies were the following: rabbit anti-EphA4 (Abcam, Cambridge, England, 1:1000), rabbit anti-MAG antibody (Invitrogen, 1:200), chicken anti-Myelin Protein Zero (Novus, CO, USA, 1:200), mouse anti-EGR2 (Krox-20) (Antibodies-online, Aachen, Germany, 1:500), and mouse anti-GAPDH (Sigma, 1:2000).

### Immunocytochemistry and Immunohistochemistry

Immunocytochemistry was performed as previously described (Gu et al., 2014). In brief, cells were fixed with 4% paraformaldehyde (PFA) for 20 min, then blocked with 5% normal donkey serum plus 0.1% Triton X-100 in PBS and incubated with primary antibodies (appropriate dilution) overnight at 4°C, followed by incubation with tetraethyl rhodamine isothiocyanate (TRITC) or Fluorescein-isothiocyanate (FITC)-conjugated secondary antibodies (Jackson Immuno Research, West Grove, PA, 1:500) at room temperature for 1 h. The cells were counterstained with Hoechst 33342 prior to visualization under a Zeiss fluorescence microscope and images were captured with a CCD Spot camera.

For immunohistochemistry, the animals were deeply anesthetized and perfused through the ascending aorta with PBS and 4% PFA. DRGs or sciatic nerves were then harvested and post-fixed in the same fixative overnight. Tissue sections (12 μm) were cut with a cryostat and processed for immunohistochemistry in a manner similar to the immunocytochemistry protocol.

The primary antibodies used for immunocytochemistry or immunohistochemistry were the following: mouse anti-S100β (Sigma, 1:500), chicken anti-NF200 antibody (Chemicon,1:500), mouse anti-EphA4 (R&D, MN, USA, 1:200), rabbit anti-EphA4 (Abcam, 1:200), rabbit anti-MAG antibody (Invitrogen, 1:200), chicken anti-Myelin Protein Zero antibody (Novus, 1:200), IgG-Alex-488 (Abcam, 1:500), IgG-Cy3 (Abcam, 1:500), Hoechst (Sigma, 1 μg/mL).

### Electron Microscopic Analysis

Nerve specimens of five randomly selected rats treated with EphA4-siRNA and EphA4 overexpression vector LV-EphA4 at 4 weeks after nerve crush were examined by electron microscopy, and. Electron microscopy experiments were performed as described (Gu et al., 2014). In brief, The sections of regenerated nerves were was removed, fixed, dehydrated, embedded in Epon 812 epoxy resin, and cut into ultra-thin sections of 60 nm thickness to be stained with lead citrate and uranyl acetate. The stained sections were observed under a transmission electron microscope (JEOL Ltd., Tokyo, Japan), and images were taken from 10 random fields of each section to determine the number of myelin sheath layers, the thickness of myelin sheaths, the diameter of myelinated nerve fibers and the g-ratio of axon area to total fiber area using Image Pro Plus software (NIH Image, National Institutes of Health).

### Statistical Analysis

Results are shown as mean ± standard error of mean (SEM). Two-tailed *t*-tests were used to compare two groups, and a one-way ANOVA was used for comparing three or more groups. The difference between means was considered to be statistically significant for a *p* < 0.05. The processing software used for data analysis consisted of Microsoft Excel (Microsoft Office, DC, USA), GraphPad Prism6.0 (GraphPad Software, CA, USA) and Image J (National Institute of Mental Health, MD, USA). The drawing software used were Adobe Photoshop CS6 (Adobe Systems, CA, USA).

## Results

### Expression and Localization of EphA4 in PNS

**Figure 1:**
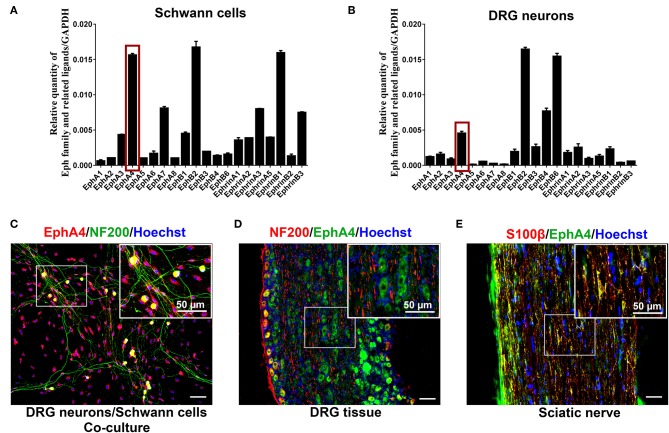
EphA4 expression in SCs and DRG neurons. **(A,B)** Relative expression of Eph family and its related ligands in DRG neurons and SCs (*n* = 3–5). The red box indicates the expression of EphA4. **(C–E)** Double labeling confirmed the presence of EphA4 in co-culture of DRG neurons and SCs **(C)**, DRG tissues **(D)** and sciatic nerve **(E)**. Boxed insets are high magnification pictures that showed the expression of EphA4 in cell body of DRG neurons or SCs. Hoechst (blue) marked the cell nucleus. Scale bar, 50 μm.

Initially, we quantified the expression of Eph family receptors and their related ligands in SCs, DRG neurons, and sciatic nerve lesion from days 1 through 28 post nerve crush using RT-qPCR. The results showed that, compared to other Eph family receptors, the *EphA4* gene was highly expressed in SCs (Figure 1A) and DRG neurons (Figure 1B), and EphA4 shows similar expression pattern with Krox20/Egr2 (a transcription factor regulating myelination) in the nerve injury model (Supplementary Figure 1). We therefore focused on EphA4 in this study. To further identify the expression and localization of EphA4 in PNS, double immunohistochemistry for S100β, an SC marker, or neurofilament (NF200), a neuronal marker, and EphA4 in DRG neurons, SCs, or DRG neurons and SCs co-culture system revealed that EphA4 is expressed in both the DRG neurons and SCs, mainly colocalizing with S100β and scantly colocalizing with the axonal marker NF200 (Figure 1C, Supplementary Figures 2, 3). To further confirm these results, we performed immunostaining for S100β, NF200, and EphA4 on the sciatic nerve and DRG tissue in naïve mice. As shown in Figures 1D,E, EphA4 was mainly localized in the soma of DRG neurons and whole SCs, consistent with the *in vitro* finding. These results demonstrated that EphA4 is expressed and localized in the entire SC.

### Similar Expression of EphA4 and Krox20 After Sciatic Nerve Crush

**Figure 2:**
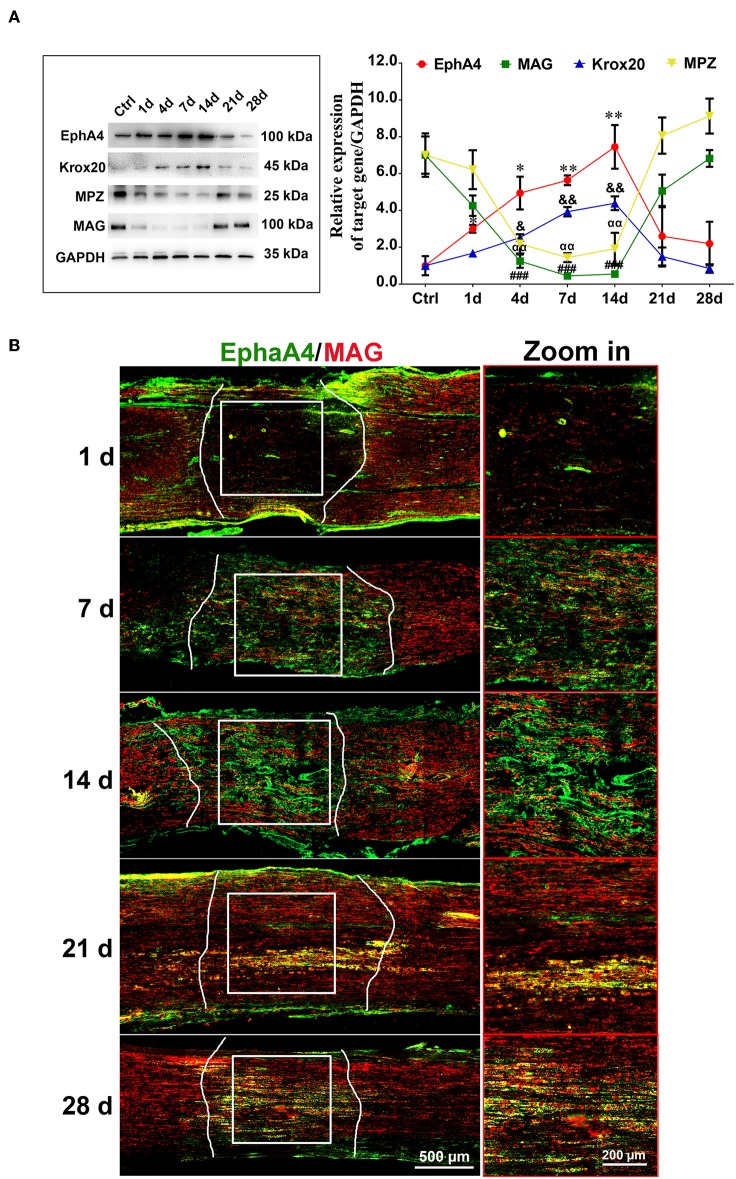
Dynamic expression of EphA4 in a rat sciatic nerve crush model. **(A)** Representative Western blot images showing EphA4, MAG, Krox-20, and MPZ levels in regenerating nerve segment at indicated different time points (i.e., 1, 4, 7, 14, 21, and 28 days) following nerve crush, and normal nerve was used as the control. Histogram compared the protein expression change of EphA4, MAG, Krox20, and P0 in regenerating nerve segment at indicated different time points following nerve crush. GAPDH served as a loading control. ^*^ or ^&^ or ^#^ or ^α^*p* < 0.05, ^**^ or ^&&^ or ^##^ or ^αα^*p* < 0.01, ^###^*p* < 0.001 vs. control, one-way ANOVA, *n* = 3 rats/group. The symbols, as ^*^, ^&^, ^#^ and ^α^, represent the EphA4, Krox-20, MAG and P0, respectively. **(B)** Immunofluorescence labeling for S100β (red) and EphA4 (green) in the injured sciatic nerve sections of rats showing the colocalization of EphA4 and S100β from day 7 of injury, indicating that EphA4 was expressed by Schwann cells from day 7 after nerve injury. Scale bar, 500 μm. Boxed insets are high magnification pictures, Scale bar, 200 μm. The representative immunohistochemical images were selected from three independent experiments.

To determine the potential role of EphA4 in SCs' control of remyelination *in vivo*, we examined its expression in sciatic nerve lesion from days 1 through 28 post-nerve crush by Western blotting, assessing levels of Krox20, MAG (myelin-associated protein) and MPZ (myelin protein zero, P0), all of which are involved in myelin formation. As shown in Figure 2A, EphA4 and Krox20 showed similar dynamics, with a progressive increase in relative expression up to 14 days, followed by a drastic decrease up to 28 days after nerve injury, while in sham group, the expression of EphA4 was stable (Supplementary Figure 4). Following an entirely different pattern, MAG and MPZ expression decreased from days 1 through 14 and then drastically increased up to 28 days. Similarly, an increase in EphA4 expression was observed at day 7, lasting until 14 days following nerve injury, as shown by double immunochemistry for EphA4 and MAG in the injured sciatic nerve sections (Figure 2B). In contrast, the expression of MAG first decreased at day 1, reaching a nadir at day 14 and rising again to reach peak expression levels at day 28. Notably, colocalization of EphA4 and MAG is not always observed because of the axon demyelination and remyelination at the site of nerve injury. Demyelination is well-known to be an early post-nerve-crush phenomenon; SCs dedifferentiate and proliferate to help clear the myelin debris. Therefore, EphA4 is not co-located with MAG at this stage, possibly because of the myelin sheath decomposition and clearance. With the axon regeneration, SCs are redifferentiated to form new myelin sheaths. Consistent with this, the colocalization of MAG and EphA4 at day 14 post-nerve-crush is possibly due to SC differentiation and expression of MAG.

### Dynamic Expression of EphA4 During Myelination *in vitro*

**Figure 3:**
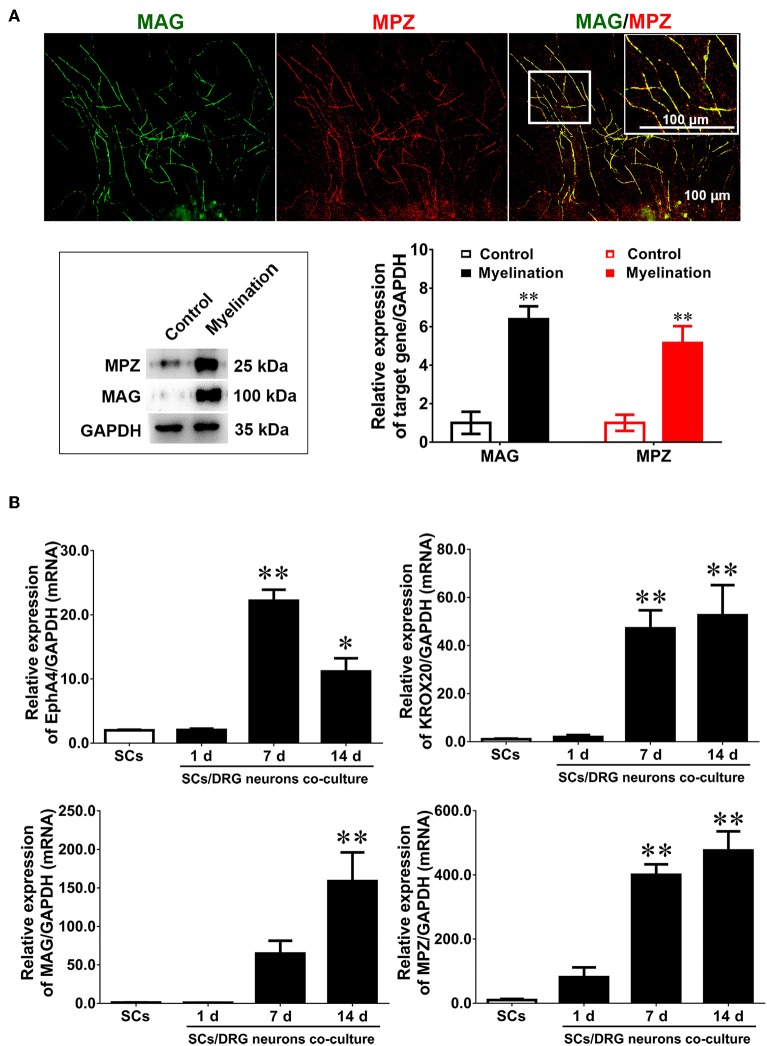
Dynamic expression of EphA4 in DRG neurons co-culture with Schwann cells. **(A)** Immunocytochemistry with anti-MAG (green) and anti-MPZ (red) displayed the myelinated axons. Also shown are the higher magnifications of the boxed areas. Scale bar, 100 μm. Western blotting was used to detect the expression of MAG and MPZ during myelination. Primary culture SCs were used as the control (*n* = 3, *t*-test, ^**^p < 0.01). **(B)** The gene expression changes of EphA4, Krox-20, MPZ, and MAG in the process of myelination (*n* = 3, one-way ANOVA, ^*^p < 0.05, ^**^p < 0.01 vs. SCs).

To investigate whether EphA4 plays a role in SC myelination, this study used a classical *in vitro* myelination model which co-cultures SCs and DRG neurons to induce myelin sheath formation (Päiväläinen et al., 2008). SCs and purified DRG tissue, surrounded by long axons after a 14 day culture period (Supplementary Figure 5), were used for co-culture experiments. After a 2 week induction into myelin, some typical segmental cords were visible, confirmed by the expression of the myelin proteins MPZ and MAG by immunocytochemistry and Western blotting (Figure 3A). Results indicated that the *in vitro* myelin model had been successfully established. We next quantified the expression levels of EphA4, Krox20, MAG, and MPZ during the process of myelination by RT-qPCR, showing that Krox20 and MAG expression levels increased concurrently with the process of myelination, while EphA4 expression increased in the early phases, decreasing at 14 days (Figure 3B). Myelination is an organized process, in which the myelin sheath is independently acquired by SC proliferation, migration, differentiation, and wrapping. Together, we proposed that EphA4 may participate in the myelination, playing different regulatory roles at different stages.

### EphA4 Knockdown Does Not Affect SC Migration

**Figure 4:**
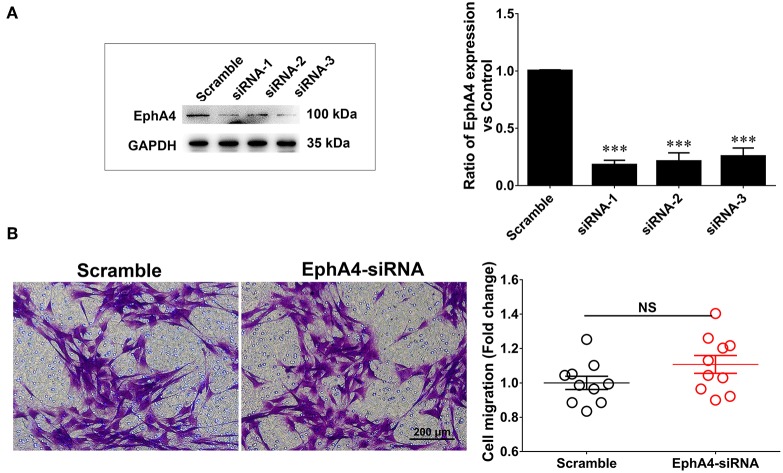
Schwann cell migration was not modulated when knockdown of EphA4 in SCs. **(A)** Transfection of SCs with EphA4 siRNA-1, siRNA-2, or siRNA-3 down-regulated EphA4 expression at the protein and mRNA levels compared with transfection of fibroblasts with negative control (Scramble) (*n* = 3, *t*-test, ^***^p < 0.001). **(B)** A transwell migration assay showed that transfection of SCs with EphA4 siRNA-3 have no effect on SC migration compared with transfection with negative control (*n* = 10, *t*-test, ^NC^*p* = 0.4451 vs. Scramble).

EphA4 has been shown to be involved in modulating cell migration in various other cell types. To investigate whether EphA4 affected SC migration, we first screened the EphA4 siRNAs, validating their knockdown efficacy by Western blotting (Figure 4A). Next, the primary cultured SCs were transfected with an EphA4 siRNA or non-targeting negative control and subjected to a transwell-based migration assay, followed by crystal violet staining and cell counting. The number of cells passing through the transwell insert membrane was not significantly different between SCs which had been transfected with EphA4-siRNA compared to that of the control group (Figure 4), suggesting that EphA4 was not likely to have any effect on SC migration.

### Reduced EphA4 Expression Increases SC Proliferation in SC/DRG Neuron Co-culture

**Figure 5:**
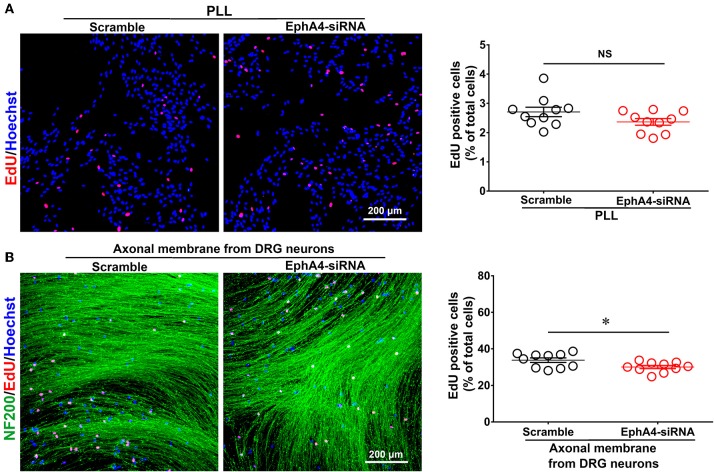
Schwann cell proliferation decreased when knockdown of EphA4 in SCs. Schwann cell seeded on PDL **(A)** or fasciculated DRG axons **(B)** were transfected with EphA4-siRNA or negative control (Scramble), and the ratio of proliferation was measured, red dots showed the proliferating SCs, blue dots showed the total cell nucleus, and NF200 immunostaining showed the fasciculated DRG axons (green), scale bar, 200 μm. Histograms showing that EphA4 knockdown could not affect SC proliferation on PDL, by contrast, the ratio of proliferation of SC cultured on fasciculated DRG axons was significantly increased after transfection with EphA4-siRNA compared with transfection with negative control (*n* = 10, *t*-test, ^*^p < 0.05).

We evaluated the effect of EphA4 on the proliferation of SCs cultured alone or co-cultured with DRG neurons. SCs were transfected with EphA4-siRNA or a negative control. Proliferation rate was estimated by EdU labeling as a proliferation assay. In isolated SCs, the number of cells double-labeled with EdU and Hoechst was not significantly affected by EphA4 knockdown (Figure 5A). In co-culture, however, the proportion of proliferative cells among SCs which had been transfected with EphA4-siRNA was significantly lower than in SCs transfected with a negative control (Figure 5B). Also, we found the SC proliferation was not significantly changed by EphA4 knockdown when treated with the neuron-conditioned medium (Supplementary Figure 6). These data indicate that EphA4 may act as a positive regulator of SC proliferation, but that this effect is only seen in co-culture conditions.

### EphA4 Antagonizes SC Differentiation *in vitro*

**Figure 6:**
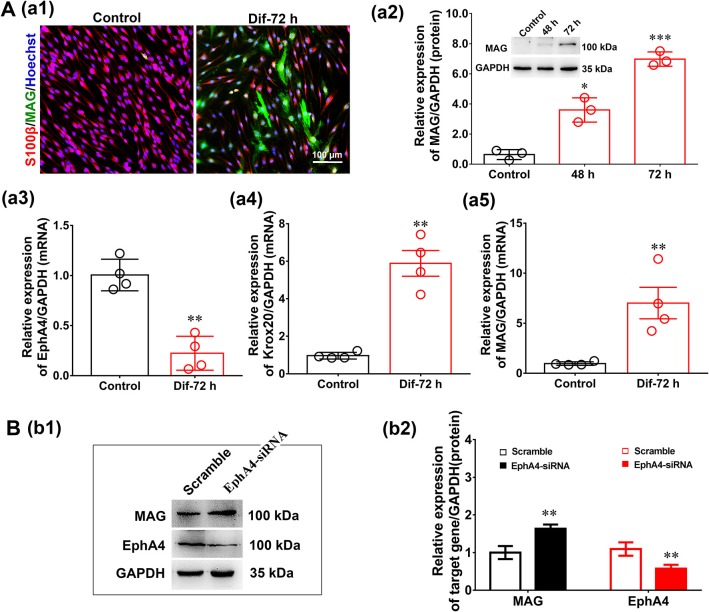
EphA4 knockdown accelerated Schwann cell differentiation. **(Aa1)** Immunocytochemistry with anti-MAG (green) displayed the SC differentiation efficacy of the different inducers that were cultured in vehicle or with dB CAMP treatment. Scale bar, 100 μm. **(Aa2)** Western blotting compared the MAG expression in differentiated cells treated with dB CAMP for 48 and 72 h, and vehicle as control. Also shown (Insert) are representative western blotting images, in which GAPDH served as an internal standard. ^*^p < 0.05, ^***^p < 0.001, vs. vehicle, *t*-test, *n* = 3 cultures per group. **(Aa3–a5)** The gene expression changes of EphA4 **(a3)**, Krox-20 **(a4)** and MAG **(a5)** during SC differentiation (*n* = 3, *t*-test, ^**^p < 0.01 vs. control). **(B)** Western blot showing EphA4 and MAG in differentiated SCs following knockdown of EphA4 in SCs. Histograms showing that EphA4 knockdown increase the expression of MAG compared with transfection with negative control (*n* = 3, *t*-test, ^**^p < 0.01).

The influence of EphA4 on SC differentiation was examined in a SC *in vitro* differentiation assay. To induce SC differentiation, we used 1 mM db-cAMP and 20 ng/ml HRG to promote differentiation by activating cyclic adenosine monophosphate (c-AMP) signaling (Pearse et al., 2004; Monje et al., 2010). Following 3 days of induction, SCs become flattened, the cytoplasm became vacuolated, and the expression of markers typically associated with myelinating SCs (e.g., MAG) increased significantly (Figures 6Aa1,a2), indicating that the SCs had reached a mature phenotype (i.e., differentiated SCs). Moreover, we also detected the expression levels of EphA4 and Krox20 by qRT-PCR during SC differentiation, finding that Krox20 expression increased, while that of EphA4 decreased (Figures 6Aa3–a5). To further characterize the effects of EphA4 on SC differentiation, SCs were transfected with EphA4-siRNA or a negative control, and the expression of MAG and EphA4 was assessed following stimulation. Results showed that the EphA4-knockdown SCs exhibited higher MAG expression levels during the process of differentiation compared to those of the control group (Figures 6Bb1,b2). Together, these results suggest that EphA4 restricted myelin formation by inhibiting SC differentiation and promoting SC proliferation rather than migration.

### EphA4 Negatively Regulates Remyelination in Regenerated Sciatic Nerves

**Figure 7:**
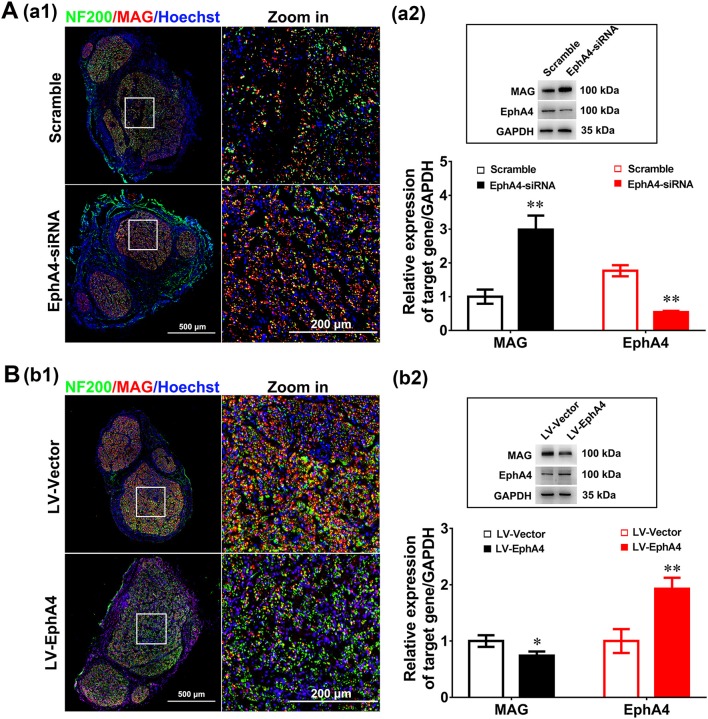
EphA4 negatively regulates myelination *in vivo*. **(Aa1,Bb1)** Double immunostaining with anti-NF200 (green) and anti-MAG (red) displayed the axon remyelination in regenerated nerves that were treated with EphA4-siRNA **(Aa1)** or overexpression vector LV-EphA4 **(Bb1)**. Also shown are the higher magnifications of the boxed areas. Scale bar, 500 μm, zoom in, 200 μm. **(Aa2,Bb2)** Western blotting compared the MAG expression in nerves treated with EphA4-siRNA **(Aa2)** or overexpression vector LV-EphA4 **(Bb2)** for 4 weeks. Also shown (Insert) are representative western blotting images, in which GAPDH served as an internal standard. ^**^p < 0.01, vs. scramble **(Aa2)**. ^*^p < 0.05, ^**^p < 0.01, vs. LV-Vector **(Bb2)**, *t*-test, *n* = 3 per group.

**Figure 8:**
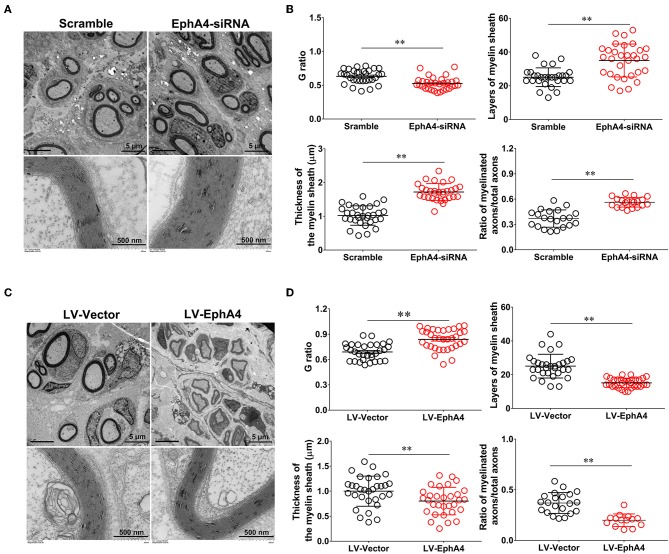
EphA4 negatively regulates myelination *in vivo*. **(A,C)** Representative transmission electron micrographs, obtained at 4 weeks post-surgery, of regenerated nerve in the rats treated with EphA4-siRNA **(A)** and EphA4 overexpression vector LV-EphA4 **(C)**. Scale bar, 5 μm, 500 nm. **(B,D)** Histograms comparing G ratio, the number of myelin sheath layers, the thickness of the myelin sheath and the ratio of myelinated axons among the rats treated with EphA4-siRNA **(B)** and EphA4 overexpression vector LV-EphA4 **(D)**. ^**^p < 0.01, vs. scramble **(B)**. ^**^p < 0.01, vs. LV-Vector **(D)**, *t*-test, *n* = 3 per group.

To determine the *in vivo* effects of EphA4 on SC remyelination during peripheral nerve regeneration, we constructed a lentivirus vector, EphA4-LV-EGFP (Ubi-MCS-3FLAG-SV40-puromycin), containing EGFP and EphA4 (NM_316539) genes and a cholesterol-modified EphA4-siRNA with higher transfection efficiency and prolonged action time by restriction of siRNA degradation (Barbara and Katarzyna, 2006; Zhao et al., 2012). To confirm the validity of the siRNA and virus transfection system, the EGFP-siRNA blank control was injected into the site of the injured or normal sciatic nerve in rats and the expression level of EGFP was assessed 3 days following transfection. As shown in Supplementary Figure 7, green fluorescence was widely observed in the normal sciatic nerve, while fluorescence was limited to the injured part of the injured nerve. Adult rats were next subjected to a sciatic nerve crush injury, immediately after which the EphA4-LV-EGFP vector or cholesterol modified EphA4-siRNA was injected into the sciatic nerve. MAG and EphA4 expression levels were assessed in the crushed sciatic nerve by immunochemistry and Western blotting at 28 days post-viral infection or siRNA interference. As shown in Figure 7A, the EphA4-siRNA interference group had higher MAG expression levels compared those of the control group. In contrast, MAG expression levels in the over-expression group were reduced compared to those in the control group (Figure 7B). To further confirm the effects of EphA4 on myelin regeneration, we used electron microscopy to observe the distribution of myelin sheaths across these four groups 4 weeks following nerve injury. The myelinated nerve fibers were characterized by a clear, electron-dense myelin sheath and perfect SC basal membrane (Figures 8A,C). Quantitative analysis revealed that the number of myelin sheath layers, the thickness of the myelin sheath, and the ratio of myelinated axons/total axons were greater in the EphA4-siRNA interference group than in the control group, and that the G ratio (a highly reliable ratio for assessing axonal myelination) was significantly reduced by EphA4 knockdown (Figure 8B). Consistent with these results, EphA4 over-expression yielded precisely opposite results, with the number of myelin sheath layers, the thickness of the myelin sheath, and the ratio of myelinated axons/total axons having all decreased, and the G ratio increased, compared to that of the control group (Figure 8D). Together, these results suggest that EphA4 negatively regulates remyelination *in vivo*.

## Discussion

We have identified a negative regulator of Schwann cell differentiation and peripheral myelination, the Eph receptor tyrosine kinase family member EphA4 receptor. In contrast with other Eph receptors, EphA4 is the most robustly distributed Eph receptor subtype and has the unique ability to bind most ephrinA and ephrinB ligands. In this study, we found that, in the Eph/Ephrin system, EphA4 was more highly expressed in DRG neurons and SCs both *in vivo* and *in vitro*, consistent with previous studies (Moss et al., 2010; Wang et al., 2013). It was recently found that Ephrin-A1-EphA4 signaling negatively regulates myelination in the CNS (Harboe et al., 2018). However, to our knowledge, no study to date has focused on the role of EphA4 in myelin formation in SCs. This study's results have revealed that, following sciatic nerve crush in rats, a 4 week treatment with a cholesterol modified EphA4-siRNA was largely effective in enhancing axonal remyelination. On the contrary, administration of EphA4-LV-EGFP inhibited remyelination, delaying it by 4 weeks. This may suggest that EphA4 signaling negatively regulates the formation of myelin sheath in SCs in the PNS in a manner similar to that of the CNS.

To determine the possible function of EphA4 in myelination, we firstly stained frozen sections of the injury segment at different time points following nerve crush. EphA4 was detected as early as 1 d following injury, with 80% of all cells stained for it at day 7. On day 21 following injury, when remyelination is at its peak, only about 10% of cells expressed EphA4, and EphA4 was almost entirely absent from distal stumps 28 d following injury. It is interesting to note that EphA receptors were found to be upregulated in active lesions of multiple sclerosis patients (Sobel, 2005), suggesting that EphA4 upregulation is associated with demyelination. Therefore, we speculated that the high expression of EphA4 in early stages of nerve injury may be related to the demyelination and dedifferentiation of SCs at this phase. We also examined expression levels of Krox20, MAG, and MPZ by Western blotting, finding that EphA4 expression was negatively correlated with MAG and MPZ expression but positively correlated with Krox20 expression (Figure 2). It is well-known that Krox20 is a key transcription factor associated with myelin formation, and that its stable expression is specifically associated with myelination in the PNS (Topilko et al., 1994). Both MPZ and MAG are essential components of the myelin sheath, expressed by myelinated glial cells and involved in the formation and maintenance of myelin (Yin et al., 1998). On the other hand, Krox-20 is a direct transcriptional activator of EphA4 (Theil et al., 1998). Based on the similar expression trends of EphA4 and Krox20 and partial colocalization with MAG, we concluded that, in PNS, EphA4 is involved in remyelination following acute nerve injury. Further, we tested the expression levels of EphA4, Krox20, MAG, and MPZ in the co-culture systems consisting of DRG explants and Schwann cells, widely used to study myelination *in vitro* (Einheber et al., 1993; Päiväläinen et al., 2008). Results demonstrated that EphA4 expression decreased with the increase in MAG expression during myelin sheath formation (Figure 3), further confirming that EphA4 may inhibit myelin sheath formation.

As is well-known, SCs can produce insulating myelin sheaths around larger axons at a one-to-one ratio in a very complex manner, involving a number of biological processes associated with SCs, including proliferation, migration, and differentiation (Morgan et al., 1991). Therefore, we further explored what type of SC biological behaviors may be affected by EphA4 to influence myelin formation. To this end, we used an EphA4 knockdown approach in SCs, characterizing changes in SC proliferation, migration, and differentiation. Our results indicated that knockdown of EphA4 had no effect on SC migration when compared to cells treated with control siRNA (Figure 4). Studies addressing the role of EphA4 in cell migration have shown that EphA4 inhibits it (Wang et al., 2013). Although these results seem to conflict with our findings, this difference may be attributed to the fact that cell migration assays vary tremendously depending on cell type, cell purity, cell density and cell culture conditions.

The EdU labeling/detection kit is a reliable and highly reproducible assay that allows for the examination of the effects of EphA4 on Schwann cell proliferation. Our study revealed that EphA4 knockdown had no effect on proliferation of SCs plated on PLL, but increased the proliferation of SCs cultured on fasciculated DRG axons when compared with cells treated with control siRNA (Figure 5). EphA4 exerts it biological function by binding to the ephrin ligands. EphA4-ephrin signaling is known to regulate cellular interactions in diverse developmental and pathological processes, including axonal guidance and synapse formation, by mediating bidirectional signaling between two relatively independent cells or cell and matrix focal adhesions (Dottori et al., 1998; Klein, 2004, 2009; Tubbs et al., 2010; Goldshmit et al., 2011; Linneberg et al., 2015). Therefore, EphA4 enhances the proliferation of SCs in co-culture conditions, possibly via axon-SC interactions. DRG neurons may release different types of cytokines and neurotrophins in the co-culture system, with these factors compensating for the role of EphA4 (Supplementary Figure 6).

An interesting finding of our study is that EphA4 can inhibit SC differentiation (Figure 6). Previous studies have demonstrated that SC switches from a non-myelinating proliferative phase to a non-proliferative myelination mode are vital to the process of myelination (Jessen and Mirsky, 2010; Salzer, 2015). EphA4 regulation of SC myelination may therefore be involved in the timing of when SCs will form permanent contacts with a given axon. Combined with the results of *in vitro* experiments, we believe that EphA4 abrogates the formation of the myelin sheath by promoting the proliferation of SCs in early stages and inhibiting the differentiation of SCs at later stages.

To further investigate the actions of EphA4 on remyelination, we injected cholesterol-modified EphA4-siRNA and an EphA4 overexpressing lentivirus into the site of injury of a crushed nerve in a rat model for 28 days, finding that EphA4 knockdown enhanced myelination by SCs and that overexpression of EphA4 in nerves decreased myelination (Figure 7). Moreover, we quantified the number of myelin sheaths formed by each SC following EphA4 knockdown or overexpression to confirm that the EphA4 signaling pathway either enhances or inhibits myelination by increasing or decreasing the myelinating capacity of SCs. Treatment with EphA4-siRNA caused a marked increase in the number of myelin sheaths formed by each cell compared with controls. In contrast, EphA4 overexpression decreased the number of myelin sheaths per cell (Figure 8) in a manner similar to that which has been observed previously following EphA4 knockdown in CNS oligodendrocytes (Harboe et al., 2018). These results suggest that EphA4 is involved in controlling SC remyelination following nerve injury.

Nonetheless, there remain a number of issues that warrant further clarification. EphA4 binding to ephrins results in cell-cell adhesion, which in turn affects the formation of synapses, cell migration, and so on. In the present study, we reported that EphA4 was involved in controlling SC proliferation and morphological differentiation, making it possible to interfere with axon-glia interactions to inhibit remyelination. However, we do not assess whether more ephrins, and which ones, bind to activate one or more downstream signaling pathways that regulate myelin sheath formation. Future studies are required to reveal (1) whether there exist ephrins specific to neurons which may be able to (2) bind EphA4 on the surface of SCs to produce or combine certain attractive and repulsive cues, and (3) to what extent these function together with neuronal activity to regulate myelination.

Taken together, our data showed that EphA4 is expressed in SCs, and that it negatively regulates the ability of SCs to undergo morphological differentiation in culture and interact with neurons during the myelination or remyelination. We suggest that EphA4 plays different roles in myelination, including the early promotion of SC proliferation followed by the inhibition of myelin formation by restricting SC differentiation. Overall, our results suggest that EphA4 may be considered as a potential target for clinically relevant myelin diseases.

## Data Availability Statement

All datasets generated for this study are included in the article/Supplementary Material.

## Ethics Statement

The animal study was reviewed and approved by the Administration Committee of Experimental Animals, Jiangsu Province, China.

## Author Contributions

YG and SL conceived and designed the experiments. RC and XY contributed to data creation and collection and analysis. BZ repeated the experiments and improved the datas. SB and SW assisted in animal experiments. RC and YG prepared the manuscript. All authors contributed to manuscript revision and read and approved of the submitted version.

### Conflict of Interest

The authors declare that the research was conducted in the absence of any commercial or financial relationships that could be construed as a potential conflict of interest.

## Supplementary Material

Supplementary Figure 1 Relative expression of Eph family and its related ligands in a rat sciatic nerve crush model. Histogram compared the mRNA expression change of EphA4, EphA7, EphB2, EphB4, EphB6, EphrinA3, EphrinB1, EphrinB3, and Krox20 in regenerating nerve segment at indicated different time points following nerve crush. GAPDH served as a control. ^**^*p* < 0.01 vs. control, one-way ANOVA, *n* = 3 rats/group.

Supplementary Figure 2 The expression of EphA4 in DRG neurons. Immunocytochemistry with NF200 (green) and EphA4 (red) of DRG neuron, and hoechst (blue) labeled nuclei. Scale bar, 50 μm.

Supplementary Figure 3 The expression of EphA4 in SCs. Immunocytochemistry with S100β (red) and EphA4 (green) of SCs, and hoechst (blue) labeled nuclei. Scale bar, 50 μm.

Supplementary Figure 4 Expression of EphA4 in sham group. Representative Western blot images showing EphA4 expression in nerve segment at indicated different time points (i.e., 1, 4, 7, 14, 21, and 28 days), and normal nerve was used as the control.

Supplementary Figure 5 Culture and purification of DRG tissues. Immunocytochemistry with S100β (red) and NF200 (green) of DRGs before and after purification, and hoechst (blue) labeled nuclei. Also shown are the higher magnifications of the boxed areas. Scale bar, 1,000 μm, zoom in, 250 μm.

Supplementary Figure 6 Schwann cell proliferation decreased when knockdown of EphA4 in SCs. After SCs were transfected with EphA4-siRNA or negative control (Scramble) for 24 h, and then cultured with DRG neuron-conditioned medium (Neuron-CM-treated) or plain medium (BM-treated). The ratio of proliferation was measured, red dots showed the proliferating SCs, and blue dots showed the total cell nucleus, scale bar, 200 μm. Histograms showing that the cell proliferation rate of SCs (transfected with EphA4-siRNA) cultured with DRG neuron-conditioned medium was not significantly different from that cultured in plain medium; by contrast, SCs (transfected with scramble) displayed an increase cultured with DRG neuron-conditioned medium (*n* = 3, *t*-test, ^*^*p* < 0.05). And the result also showed that the significant difference between SCs (transfected with scramble) and SCs (transfected with EphA4-siRNA) cultured in DRG neuron-conditioned medium, but no difference in plain medium (*n* = 3, *t*-test, ^#^*p* < 0.05).

Supplementary Figure 7 EGFP (green, transfection) in the slice of the sciatic nerve after 3 days of EGFP-siRNA transfection. Also shown are the higher magnifications of the boxed areas. Scale bar, 50 μm, zoom in, 200 μm. The figure displayed that the green fluorescence dots are widely expressed in the normal sciatic nerve, while in the injured nerve, the fluorescence was distributed only in the injured part of the nerve.

Supplementary Table 1 RT-PCR primers.

Supplementary Table 2 EphA4-siRNA sequence.
