# Supplementary figures and images for: EphA4 Negatively Regulates Myelination by Inhibiting Schwann Cell Differentiation in the Peripheral Nervous System
Source: Front Neurosci. 2019 Nov 13;13:1191. doi: 10.3389/fnins.2019.01191 (PMC6863774; doi:10.3389/fnins.2019.01191)

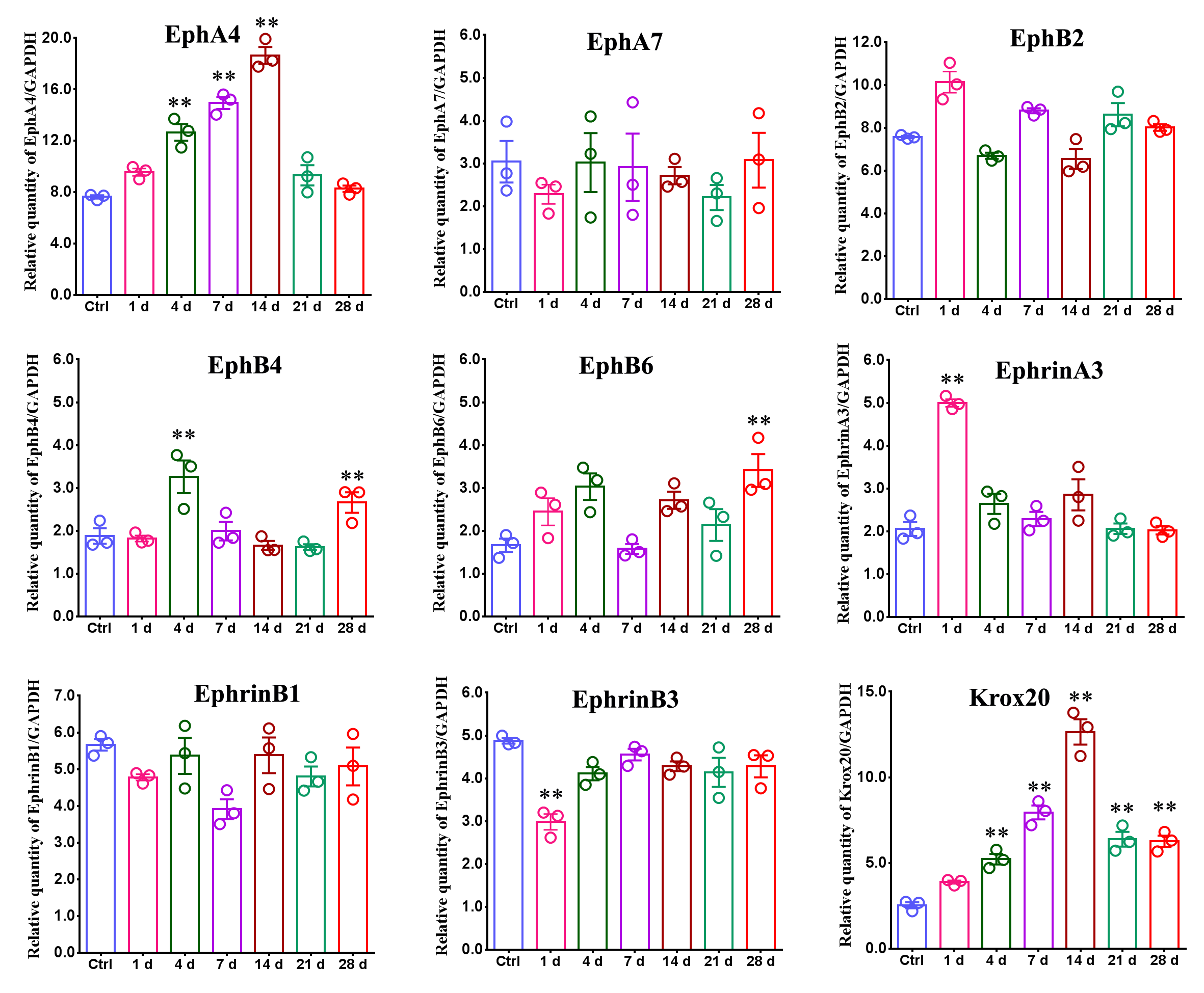

Supplement: Supplementary Figure 1 — Relative expression of Eph family and its related ligands in a rat sciatic nerve crush model. Histogram compared the mRNA expression change of EphA4, EphA7, EphB2, EphB4, EphB6, EphrinA3, EphrinB1, EphrinB3, and Krox20 in regenerating nerve segment at indicated different time points following nerve crush. GAPDH served as a control. **p < 0.01 vs. control, one-way ANOVA, n = 3 rats/group. [file Image_1.tif]

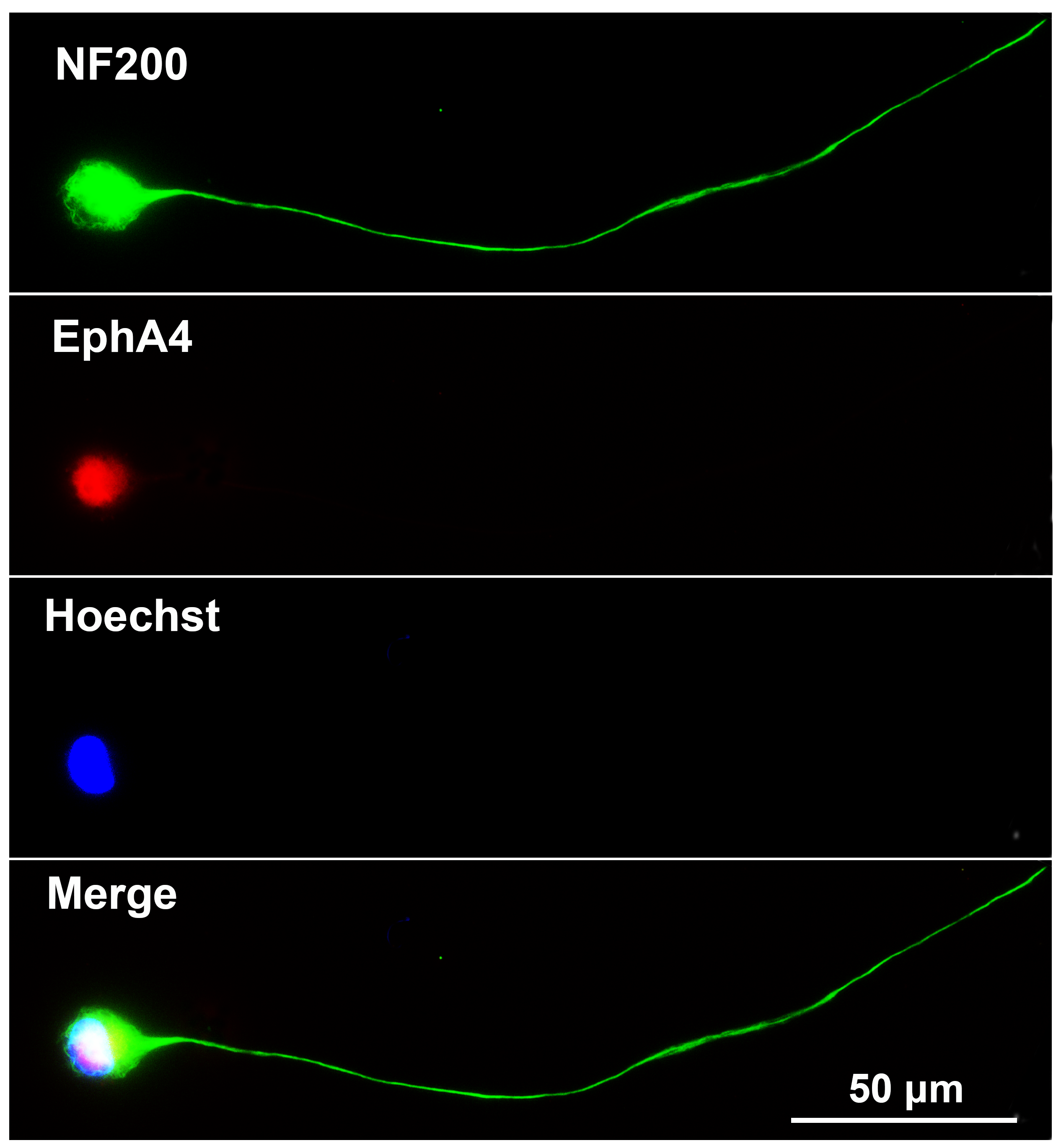

Supplement: Supplementary Figure 2 — The expression of EphA4 in DRG neurons. Immunocytochemistry with NF200 (green) and EphA4 (red) of DRG neuron, and hoechst (blue) labeled nuclei. Scale bar, 50 μm. [file Image_2.tif]

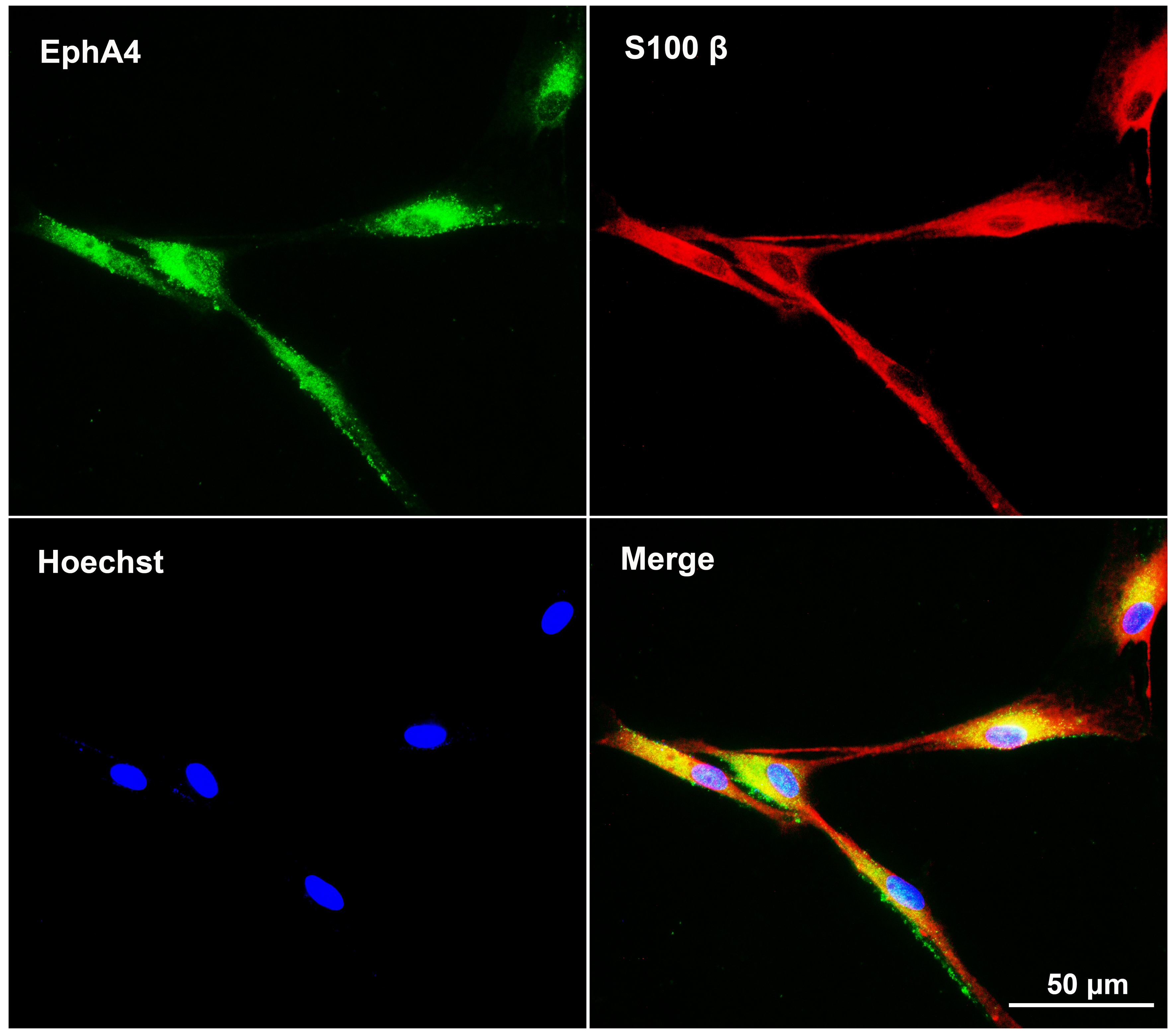

Supplement: Supplementary Figure 3 — The expression of EphA4 in SCs. Immunocytochemistry with S100β (red) and EphA4 (green) of SCs, and hoechst (blue) labeled nuclei. Scale bar, 50 μm. [file Image_3.tif]

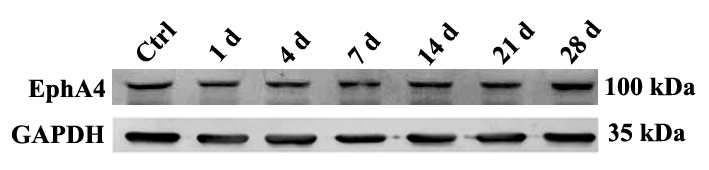

Supplement: Supplementary Figure 4 — Expression of EphA4 in sham group. Representative Western blot images showing EphA4 expression in nerve segment at indicated different time points (i.e., 1, 4, 7, 14, 21, and 28 days), and normal nerve was used as the control. [file Image_4.tif]

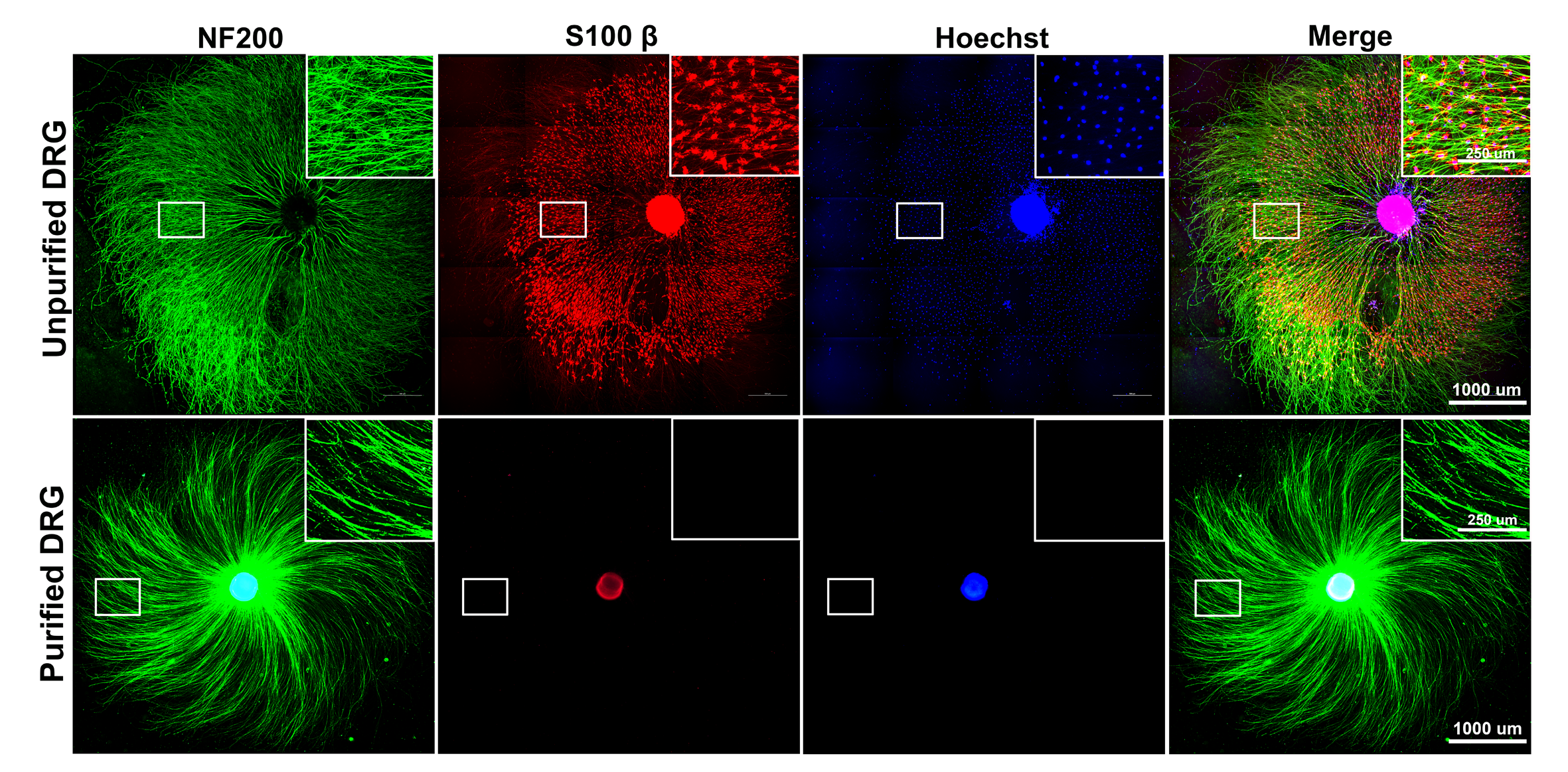

Supplement: Supplementary Figure 5 — Culture and purification of DRG tissues. Immunocytochemistry with S100β (red) and NF200 (green) of DRGs before and after purification, and hoechst (blue) labeled nuclei. Also shown are the higher magnifications of the boxed areas. Scale bar, 1,000 μm, zoom in, 250 μm. [file Image_5.tif]

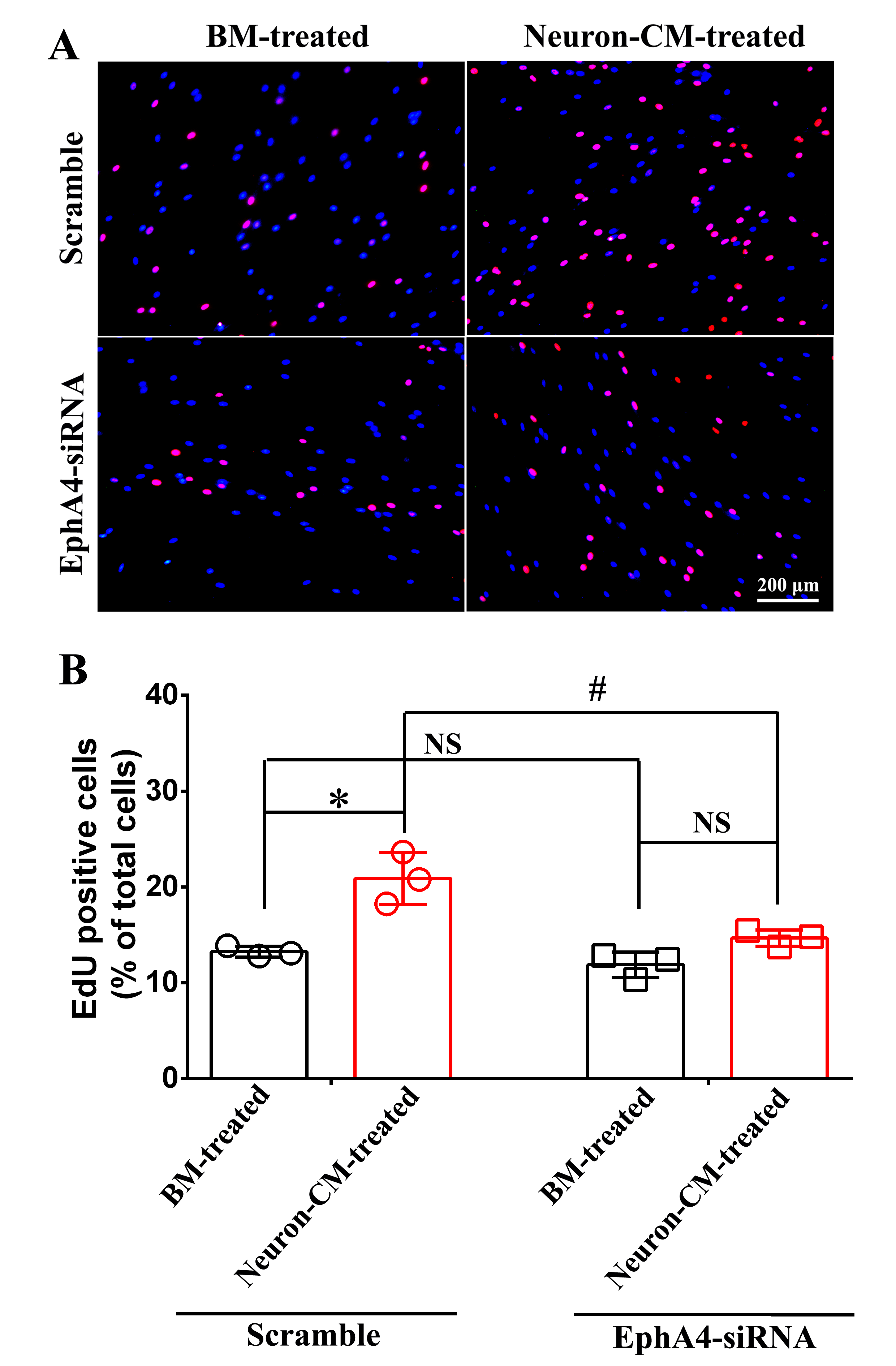

Supplement: Supplementary Figure 6 — Schwann cell proliferation decreased when knockdown of EphA4 in SCs. After SCs were transfected with EphA4-siRNA or negative control (Scramble) for 24 h, and then cultured with DRG neuron-conditioned medium (Neuron-CM-treated) or plain medium (BM-treated). The ratio of proliferation was measured, red dots showed the proliferating SCs, and blue dots showed the total cell nucleus, scale bar, 200 μm. Histograms showing that the cell proliferation rate of SCs (transfected with EphA4-siRNA) cultured with DRG neuron-conditioned medium was not significantly different from that cultured in plain medium; by contrast, SCs (transfected with scramble) displayed an increase cultured with DRG neuron-conditioned medium (n = 3, t-test, *p < 0.05). And the result also showed that the significant difference between SCs (transfected with scramble) and SCs (transfected with EphA4-siRNA) cultured in DRG neuron-conditioned medium, but no difference in plain medium (n = 3, t-test, #p < 0.05). [file Image_6.tif]

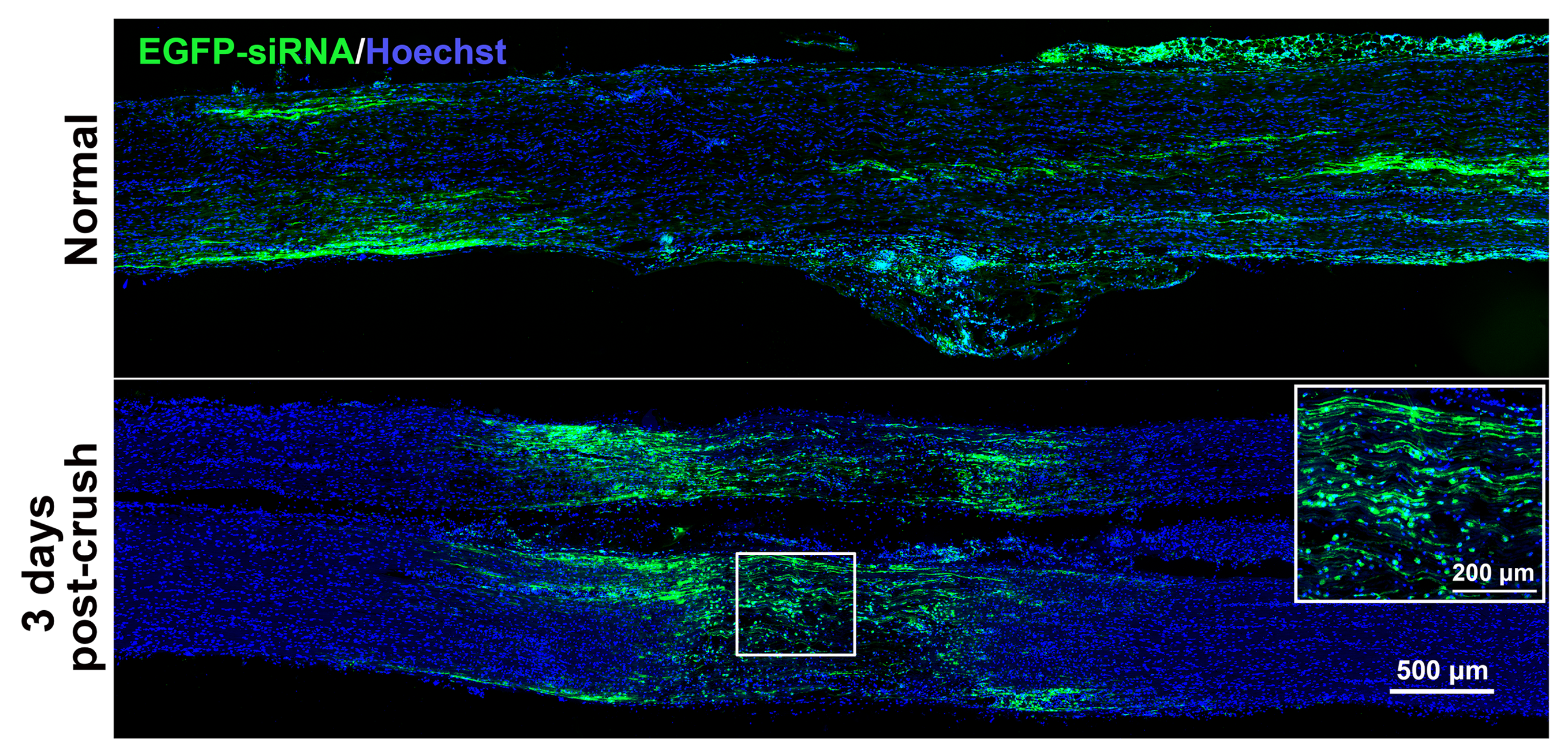

Supplement: Supplementary Figure 7 — EGFP (green, transfection) in the slice of the sciatic nerve after 3 days of EGFP-siRNA transfection. Also shown are the higher magnifications of the boxed areas. Scale bar, 50 μm, zoom in, 200 μm. The figure displayed that the green fluorescence dots are widely expressed in the normal sciatic nerve, while in the injured nerve, the fluorescence was distributed only in the injured part of the nerve. [file Image_7.tif]
