# Supplementary material for: EphA4 Negatively Regulates Myelination by Inhibiting Schwann Cell Differentiation in the Peripheral Nervous System
Source: Front Neurosci. 2019 Nov 13;13:1191. doi: 10.3389/fnins.2019.01191 (PMC6863774; doi:10.3389/fnins.2019.01191)
Supplement: Supplementary Table 1 — RT-PCR primers. [file Table_1.docx]

**Supplementary Table.1. RT-PCR primers**

| **Gene** | **Forward primers** | **Reverse primers** | **Product size** |
| --- | --- | --- | --- |
| EphA1 | F: CTGCACAGGGAGCCTTAGAC | R: AACTGGCCCATGATAGTTGC | 217 bp |
| EphA2 | F: GGGGATGCCAATAGCTACAA | R: TGGATGAAGAAGCCAATTCC | 248 bp |
| EphA3 | F: GCTGGCAGAAAGACAGGAAC | R: ACCGTTAAGCCAATCACCTG | 188 bp |
| EphA4 | F: TACAGTGTGGCTCTGGCTTG | R: CAAGGGTTCGCTGAAGTCTC | 222 bp |
| EphA5 | F: GACGGTGGGAGTCATCTTGT | R: ATCCTGCTTTGCTTTGCTGT | 151 bp |
| EphA6 | F: ACTGTGCGGAACTTGGCTAT | R: CAGAAAAGGAATAGGACACACCA | 226 bp |
| EphA7 | F: AGCTGACAACGGAGGAAGAA | R: CGAACGTGTAATTTGCATGG | 179 bp |
| EphA8 | F: GTGAATTTGATCTCGGCGTAGAC | R: CGGCCCATCCACACGTAT | 119 bp |
| EphB1 | F: TCAGTGGCAAGATGTGCTTC | R: GCCTGTGCTGTAATGCTGAA | 206 bp |
| EphB2 | F: CAACTGGCTACGGACCAAAT | R: TCTCCATCCAGTTGGGAAAG | 188 bp |
| EphB3 | F: CACTGTCACCAGCCAGAAGA | R: GCAGACAAGAGCAATGACCA | 247 bp |
| EphB4 | F: CACTGACCGTGAAGTGCCTC | R: GCGCCCTTCTCGTGATACTT | 144 bp |
| EphB6 | F: CGAGAGGGCCAGTTCAGTAG | R: GCGAAGCAAGGAACTTGAAC | 192 bp |
| EphrinA1 | F: CTTACACTGCGGAGCTTTCGT | R: ACGGTGTAGTCCTCCTCTCG | 166 bp |
| EphrinA2 | F: GTCTACTGGAACCGCAGCAA | R: AGTGCGGGCAATAGATGTCC | 112 bp |
| EphrinA3 | F: TACATGGTGAACCTGAGCGG | R: ATGGTGAACTGGGGGAGAGT | 293 bp |
| EphrinA5 | F: AGGTGTTCGTGATCGTGTTT | R: ACGGGAGGAGACTGTGCTAT | 200 bp |
| EphrinB1 | F:   CGCATTGTTGGGAGACAACC | R:   CTTGAAGCACTCTCCTCCCG | 333 bp |
| EphrinB2 | F:   GGAGACACCGCAAACACTCT | R: CCGCTGACCTTCTCGTAGTG | 166 bp |
| EphrinB3 | F:   CTCCTCAATCCAGTCGCC | R: GCCCAGTAACCACCCCAAA | 171 bp |
| Krox-20 | F: CCCTCTCCAAAAACGGCTTC | R: CTGGGATTTTGTCTACGGCCT | 107 bp |
| MAG | F: CGTCGCCTCACTGTACTTCA | R: CCTCGAAGGCTGAGATGGAC | 226 bp |
| MPZ | F: CGTGATCGGTGGCATCCTC | R: GGCATACAGCACTGGCGTCT | 184 bp |
| GAPDH | F:   AGTGCCAGCCTCGTCTCATA | R: GATGGTGATGGGTTTCCCGT | 248 bp |
