# Supplementary material for: EphA4 Negatively Regulates Myelination by Inhibiting Schwann Cell Differentiation in the Peripheral Nervous System
Source: Front Neurosci. 2019 Nov 13;13:1191. doi: 10.3389/fnins.2019.01191 (PMC6863774; doi:10.3389/fnins.2019.01191)
Supplement: Supplementary Table 2 — EphA4-siRNA sequence. [file Table_2.docx]

**Supplementary Table.2. EphA4-siRNA sequence**

| **Name** | **Target sequence** |
| --- | --- |
| EphA4-siRNA_01 | GGAAGTAAGCATTATGGAT |
| EphA4-siRNA_02 | GCAGATGAAGAGAAGCATT |
| EphA4-siRNA_03 | GCAATCACGCACCAGAATA |
| EphA4-siRNA_01  (2OMe+5Chol) | CTGCACAGGGAGCCTTAGAC |
